# Genetic Variation in the Base Excision Repair Pathway, Environmental Risk Factors, and Colorectal Adenoma Risk

**DOI:** 10.1371/journal.pone.0071211

**Published:** 2013-08-12

**Authors:** Roman Corral, Juan Pablo Lewinger, Amit D. Joshi, A. Joan Levine, David J. Vandenberg, Robert W. Haile, Mariana C. Stern

**Affiliations:** 1 Department of Preventive Medicine, Keck School of Medicine of USC, Norris Comprehensive Cancer Center, University of Southern California, Los Angeles, California, United States of America; 2 Department of Epidemiology, Harvard School of Public Health, Boston, Massachusetts, United States of America; 3 Division of Oncology, Department of Medicine, Stanford School of Medicine, Stanford, California, United States of America; MOE Key Laboratory of Environment and Health, School of Public Health, Tongji Medical College, Huazhong University of Science and Technology, China

## Abstract

Cigarette smoking, high alcohol intake, and low dietary folate levels are risk factors for colorectal adenomas. Oxidative damage caused by these three factors can be repaired through the base excision repair pathway (BER). We hypothesized that genetic variation in BER might modify colorectal adenoma risk. In a sigmoidoscopy-based study, we examined associations between 182 haplotype tagging SNPs in 14 BER genes, and colorectal adenoma risk, and examined their potential role as modifiers of the effect cigarette smoking, alcohol intake, and dietary folate levels. Among all individuals, no statistically significant associations between BER SNPs and adenoma risk persisted after correction for multiple comparisons. However, among Asian-Pacific Islanders we observed two SNPs in *FEN1* and one in *NTHL1*, and among African-Americans one SNP in *APEX1* that were associated with colorectal adenoma risk. Significant associations were also observed between SNPs in the *NEIL2* gene and rectal adenoma risk. Three SNPS modified the effect of smoking (*MUTYH* interaction p = 0.002; *OGG1* interaction p = 0.013); *FEN1* interaction p = 0.013)), one SNP in *LIG3* modified the effect of alcohol consumption (interaction p = 0.024) and two SNPs in *LIG3* modified the effect of dietary folate (interaction p = 0.001 and p = 0.08) on colorectal adenoma risk. These findings support a role for genetic variants in the BER pathway as potential modifiers of colorectal adenoma risk. Our findings strengthen the role of oxidative damage induced by key lifestyle and dietary risk factors in colorectal adenoma formation.

## Introduction

Colorectal cancer (CRC) is the third most common cancer diagnosed in men and women and is the third leading cause of cancer death in the United States [1]. In 2011, there were approximately 141,210 individuals diagnosed with CRC and 49,380 deaths from this disease in the United States [1]. More than 80% of CRC evolve from neoplastic adenomatous polyps or adenomas [2]. Prevalence of adenomas is estimated at 30% at age 50 and 40% by age 60 [3]. Compared to small (<1 cm) adenomas, larger ones (≥ 1 cm) have a greater potential to grow and progress into CRC [4]. Use of endoscopy screening with polyp removal has significantly reduced CRC incidence and mortality [5], [6], reinforcing the pathogenetic relationship between adenomas and CRC. Therefore, the identification of risk factors for adenoma development has significant public health implications.

Cigarette smoking [7], [8] and folate deficiency [9] are established adenoma risk factors, whereas folate supplementation may promote progression of established adenomas [10]. Cigarette smoke contains reactive oxygen species (ROS) and chemical carcinogens [11] which can damage DNA [11], [12]. Additionally, smoking has been associated with decreased folate levels [13], which may contribute to folate deficiency, which is associated with double strand breaks and abasic sites [14].

Alcohol consumption is currently considered a convincing CRC risk factor [15], [16]. Increasing alcohol intake has previously been shown to reduce folate levels [17], [18]. Alcohol metabolism generates acetaldehyde, a known mutagen which generate ROS in the colon lumen [19], [20], directly inhibits O^6^ methylguanine-methyl-transferase, an enzyme involved in removal of alkylation-induced DNA damage, and leads to hyper regeneration of crypt cells which induces multiple forms of DNA damage [19], [21]. In addition, alcohol metabolism can induce cytochrome P450 enzymes, which can increase activation of chemical pro-carcinogens [19], [22], [23].

Overall, smoking, alcohol consumption and decreased folate availability can lead to oxidative DNA damage and excess uracil misincorporation, which may all contribute to adenoma development. The base excision repair (BER) pathway, is the predominant mechanism for repair of oxidative DNA damage [24]. We hypothesized that genetic variation in BER genes may modify the risk of colorectal adenomas, in particular, in combination with smoking, alcohol, and low dietary folate. We examined potential associations between single nucleotide polymorphisms (SNPs) in genes involved in BER and distal adenoma risk, and considered their role as potential modifiers of the effect of cigarette smoking, alcohol consumption, and dietary folate intake. Using data and samples from a sigmoidoscopy-based study conducted in Los Angeles County we comprehensively investigated associations between genetic variation in 14 BER genes and colorectal adenoma risk.

## Methods

### Ethics Statement

The research protocol was approved by the institutional review boards at both USC and Kaiser Permanente, and all subjects signed a written informed consent approved by both institutions.

### Study Subjects

Study participants were enrolled in a University of Southern California/Kaiser Permanente study of risk factors for colorectal adenomas. All individuals were examined by flexible sigmoidoscopy from 1991 to 1993 (phase I) and from 1993 to 1995 (phase II) at one of two southern California Kaiser Permanente clinics (Bellflower or Sunset), and were recruited using identical criteria, as we have previously described [25], [26]. Briefly, cases were individuals with a first-time diagnosis of a histologically confirmed adenoma. Controls were selected from the remaining eligible individuals who did not present with polyps at sigmoidoscopy examination and had no past history of histologically confirmed adenomas. Controls were individually matched to cases by gender, age (within 5 years), sigmoidoscopy date (within 3 months), and Kaiser Permanente clinic. All subjects signed a written informed consent approved by the Institutional Review Board, donated a blood sample, and completed two questionnaires. A risk factor questionnaire was administered during an in-person interview, and collected data on demographics, smoking history, family history of cancer, physical activity and other factors described previously [27], [28]. A semi-quantitative food frequency questionnaire was administered in reference to diet during the year before sigmoidoscopy examination, as previously described [29].

### SNP Selection and Genotyping

TagSNPs for each BER gene were selected using Haploview Tagger [30] based on the HapMap CEPH (CEU) population using the following criteria: minor allele frequency (MAF) ≥5%, pairwise r^2^≥0.95, and a distance from the closest SNP greater than 60 base pairs on the Illumina platform. Linkage disequilibrium (LD) blocks were defined using data from HapMap data release #16c.1, June 2005, on NCBI B34 assembly, dbSNP b124. For each gene, we included the 5′- and 3′-most SNP within the LD block within ∼10 kb upstream and ∼5 kb downstream. In regions of no or low LD, tagSNPs with a MAF ≥5% at a density of ∼ 1 per kb were selected from either HapMap or dbSNP. In this analysis, we report our results for 182 tagSNPs across 14 genes that participate in the BER pathway (Table 1 and Table S1). TagSNPs were genotyped on the Illumina GoldenGate platform, as we previously described [31]. Among the BER genes in our study, we required that all 182 tagSNPs have call rates ≥0.90 and that we not observe evidence of statistically significant deviations of observed from expected values, when assuming Hardy-Weinberg equilibrium ≥0.00027 using exact tests (Bonferroni-corrected p value; α = 0.05/182). All tagSNPs met these two requirements. Additionally, we required all individuals had overall call rates ≥90%, which led to exclusion from analyses of 89 individuals. We included in final analyses genotype data from 1,368 (94%; 677 cases and 691 controls) of 1,457 total subjects in this study. Demographic and matching characteristics for individuals with and without genotype data were not statistically significantly different.

**Table 1:** Base excision repair pathway genes investigated.

Gene Symbol	# tagSNPs	Chromosomal location	Protein Function
*APEX1*	13	14q11.2	Endonuclease
*FEN1*	7	11q12	Endonuclease
*LIG1*	23	19q13.2-q13.3	Ligase
*LIG3*	7	17q11.2-q12	Ligase
*MUTYH*	7	1p34.1	Glycosylase
*NEIL1*	4	15q33.33	Glycosylase
*NEIL2*	43	8p23.1	Glycosylase
*NTHL1*	9	16p13.3	Endonuclease and Glycosylase
*OGG1*	7	3p26	Glycosylase
*PARP1*	18	1q41-q42	poly(ADP-ribosyl)ation
*POLβ*	6	8p12-p11	Polymerase
*POLD1*	7	19q13.3	Polymerase
*SMUG1*	10	12q13.11–13.3	Glycosylase
*XRCC1*	21	19q13.2	Scaffolding protein

Genes are listed according to HUGO gene nomenclature format (http://www.genenames.org), Abbreviations: APEX1, APEX nuclease 1; FEN1, flap structure-specific endonuclease 1; LIG1, ligase I, DNA, ATP-dependent; LIG3, ligase III, DNA, ATP-dependent; MUTYH, mutY homolog (E. coli); NEIL1, nei endonuclease VIII-like 1 (E. coli); NEIL2, nei endonuclease VIII-like 2 (E. coli); NTHL1, nth endonuclease III-like 1 (E. coli); OGG1, 8-oxoguanine DNA glycosylase; PARP, poly (ADP-ribose) polymerase 1; POLβ, polymerase (DNA directed),beta; POLD1, polymerase (DNA directed), delta 1, catalytic subunit 125 kDa; SMUG1, single-strand-selective monofunctional uracil-DNA glycosylase 1; XRCC1, X-ray repair complementing defective repair in Chinese hamster cells 1.

### Statistical Analysis

#### SNP main effect analyses

Deviations of observed genotype frequencies from those expected when assuming HWE were examined among controls by race/ethnicity using exact tests. We used unconditional logistic regression, assuming a log-additive model, to estimate per-allele odds ratios (ORs) and corresponding 95% confidence intervals (95% CIs) for the association between genotype and adenoma risk, adjusting for race (Non-Hispanic Whites, Latinos, African-American, Asian/Pacific Islander), study phase (phase I/phase II), age at sigmoidoscopy (continuous), gender, exam date, and clinic (Bellflower or Sunset). In this study, similar results are obtained when using unconditional logistic regression adjusting for the matching factors as using conditional logistic regression, with the benefit of using all genotype information in the study population [26]. Additional control for the following adenoma risk factors did not change ORs by more than 10%; therefore, they were not included in final analyses: alcohol intake (g/day), smoking status, dietary folate intake (mcg/day), body mass index, multivitamin use (yes/no), total caloric intake, total dietary fiber (g/day).

When testing SNP main effects we corrected for multiple testing within each gene region as well as across all BER gene regions investigated. Specifically, for each tagSNP obtained under a log-additive model, p-values were corrected for multiple testing, taking into account multiple correlated tests due to LD between SNPs within each gene region, using the P_ACT_ (p-value adjusted for correlated tests) method implemented in the P_ACT_ R package [32]. Additionally, we corrected for multiple testing between gene regions by applying a Bonferroni correction multiplying each P_ACT_ p-value by the 14 investigated BER gene regions to determine overall pathway significance (p_pathway_) [33]. We assessed potential heterogeneity of SNP main effects by race/ethnicity (among tagSNPs with MAF >5% in controls for each race/ethnicity), by performing 3df likelihood-ratio tests of interaction between genotypes and race/ethnicity. We used multinomial logistic regression to examine differences in SNP main effects by adenoma location (rectal versus left colon) and adenoma size (<1 cm) versus ≥1 cm) with respect to the control group. SNP main effect p-values for stratified analyses were corrected using P_ACT_ as described above. When considering linkage between different SNPs we used the square of the correlation coefficient (R^2^) to estimate pairwise LD using Haploview [30].

#### TagSNP × environmental risk factor interactions

We investigated whether BER genes tagSNPs modify the effect of alcohol use, dietary folate intake, or smoking in all individuals and in non-Hispanic Whites (NHW) only. We categorized smoking variables using median values among smoking controls to create the following variables: smoking status (never, quit, current), years of smoking (0, ≤26 years, >26 years), pack-years smoked (0, ≤21 pack years, >21 pack years). We also considered the following alcohol intake variables: number of drinks per day (never, ≤1 drink/day, >1 drink/day) and median daily alcohol intake (using median intake among drinking controls as a cut point: 0, ≤6 g/d, >6 g/d), with one alcoholic drink per day defined as approximately 15 grams of ethanol. We considered a dietary folate intake variable (low/medium/high) defined using tertiles of dietary folate intake among controls as cut points: ≤267 mcg/day, >267–387 mcg/day, ≥388 mcg/day).

Analyses of gene-environment interactions (GxE) were conducted by testing interaction terms between tagSNPs (assuming a log-additive model) and each environmental exposure using likelihood ratio tests based on unconditional logistic regression. To reduce the multiple testing burden and avoid possible failure of asymptotic tests, any tagSNPs for which a genotypic category count was less than 10 for any of the environmental factor strata considered was not included in analyses. Gene-exposure interactions were mutually adjusted for all three exposures considered. Further adjustment for body mass index, multivitamin use (yes/no), total caloric intake, or total dietary fiber (g/day) produced almost identical estimates; therefore we did not keep these variables in the models.

We corrected for multiple testing by applying two Bonferroni corrections to the crude interaction p-values which we report separately: first, we applied a within gene region Bonferroni correction (interaction p_gene_), by considering the number of tagSNPs in its respective gene region; second, we applied an overall pathway Bonferroni correction (interaction p_pathway_), by considering the 14 investigated BER gene regions. Statistical significance was declared if either corrected p-values were <0.05. All tests conducted were two sided and all statistical analysis were conducted using Stata 11 SE (Stata Corporation, College Station, TX) and the R programming language (The R Project for Statistical Computing, http://www.r-project.org).

## Results

Demographic characteristics of all cases (N = 721) and controls (N = 736) in our study are summarized in Table 2. As we previously described [25], there were no differences in age, gender, ethnicity, alcohol intake and smoking patterns between Phase I and Phase II participants. However, Phase II participants had higher dietary folate intake than those who participated in phase I (p = <0.001). Fifty-two percent of enrolled subjects were NHW. The mean age of cases was 61.46 years (±6.75) and the mean age of controls was 61.67 years (±6.88). Cases smoked longer and more intensely than controls and were more likely to be current smokers (p<0.001). Cases were also found to have a lower mean dietary folate intake (mcg/d) and a lower mean dietary fiber intake (g/d) than controls (p = 0.013; p = 0.036). Approximately 81% of adenomas were colon adenomas and approximately 67% were small adenomas (<1 cm).

**Table 2:** Demographics and descriptive characteristics of cases and controls.

	Controls		Cases		
Variable	n = 736	%	n = 721	%	p
Mean age at interview,y (±SD)	61.46	6.75	61.67	6.88	0.550
Mean Smoking years,y (±SD)	14.6	16.77	18.52	17.86	<0.001
Mean smoking packyears, (±SD)	16.61	31.51	21.04	31.51	0.008
Mean alcohol intake, y (±SD)	8.03	14.76	9.44	18.54	0.108
Mean dietary folate, mcg/day (±SD)	366.63	210.28	340.08	194.28	0.013
Mean Calories (±SD)	2046.25	951.76	2081	924.43	0.473
Mean dietary fiber, g/day (±SD)	22.84	13.18	21.43	12.67	0.036
Mean saturated fat, g/day (±SD)	23.37	13.38	24.4	13.42	0.147
Mean BMI (±SD)	27.08	4.79	27.57	4.61	0.051
Median Age at interview,y					
≤60 yrs	331	44.97	313	43.41	0.549
>60 yrs	405	55.03	408	56.59	
Race/ethnicity					
non-Hispanic White	386	52.59	372	52.10	0.344
African-American	112	15.26	126	17.65	
Hispanic	149	20.30	123	17.23	
Asian-Pacific Islander	79	10.76	80	11.20	
Other	8	1.09	13	1.82	
Sex					
Male	479	65.17	465	64.49	0.787
Female	256	34.83	256	35.51	
Clinic of diagnosis					
Bellflower	472	64.13	464	64.36	0.929
Sunset	264	35.87	257	35.64	
Study Phase					
Phase I	460	62.50	438	60.75	0.492
Phase II	276	37.50	283	39.25	
Number of Adenomas					
1			638	88.73	
≥2			81	11.27	
Missing			2		
Adenoma site					
Rectum (<15 cm)			137	19.13	
Colon (≥15 cm)			579	80.87	
Missing			5		
Adenoma size					
Small (<1 cm)			476	66.85	
Large (≥1 cm)			236	33.15	
Missing			9		
Smoking					
Never	301	42.94	253	36.77	<0.001
Former	326	46.50	312	45.35	
Current	74	10.56	123	17.88	
Missing	35		33		
Smoking years					
Never	301	43.43	253	37.21	<0.001
>0–26 yrs	198	28.57	168	24.71	
>26 yrs	194	27.99	259	38.09	
Missing	43		41		
Smoking Pack-years					
Never	301	43.56	253	37.26	0.001
>0–21	196	28.36	171	25.18	
>21	194	28.08	255	37.56	
Alcohol intake					
Never	283	38.45	268	37.33	0.323
≤6 g/day	227	30.84	204	27.41	
>6 g/day	226	30.71	246	34.26	
Missing	0		3		
Alcohol intake					
Never	283	38.45	268	37.33	0.864
≤15 g/day	333	45.24	327	45.54	
>15 g/day−30 g/day	50	6.79	46	6.41	
>30 g/day	70	9.51	77	10.72	
Missing	0		3		
Alcohol intake					
Never	283	38.45	268	37.33	0.871
≤15 g/day	333	45.24	327	45.54	
>15 g/day	120	16.30	123	17.13	
Missing	0		3		
Dietary folate					
Low (≤267 mcg/day)	248	33.70	283	40.60	0.024
Medium (>267–387 mcg/day)	241	32.74	210	30.13	
High (≥388 mcg/day)	247	33.56	204	29.27	
Missing					
Multivitamin use					
No	303	41.28	293	42.40	0.668
Yes	431	58.72	398	57.60	
Missing	2		30		

### BER Genes and Colorectal Adenoma Risk

Among all individuals combined, out of the 182 tagSNPs only *NEIL2* rs11785481 showed a statistically significant association with adenoma risk; however, it was not statistically significant after multiple comparisons adjustment within gene region (P_ACT_) (OR = 0.70; 95%CI = 0.55–0.90; p = 0.006; P_ACT_ = 0.140) (Table 3).

**Table 3:** Colorectal adenoma SNP associations among the entire population and stratified by race/ethnicity.

Gene	SNP	All combined	Non-Hispanic White	African-American	Hispanic	Asian-Pacific Islander	P_het_ e
*NEIL2*	rs11785481						0.131
	Ca/Co^a^	666/692	354/364	121/109	116/140	75/79	
	OR b	0.70	0.64	0.60	1.29	0.25	
	95%CI	0.55–0.90	0.48–0.87	0.24–1.53	0.72–2.31	0.05–1.37	
	p^c^	0.006	0.004	0.286	0.387	0.111	
	p_ACT_ d	0.139	0.089	1.000	1.000	0.806	
*FEN1*	rs108499						0.024
	Ca/Co^a^	691/663	353/365	122/109	113/140	75/77	
	OR b	1.06	0.95	0.67	1.09	2.12	
	95%CI	0.90–1.25	0.76–1.18	0.37–1.21	0.76–1.57	1.30–3.45	
	p^c^	0.516	0.635	0.185	0.641	0.002	
	p_ACT_ d	1.000	1.000	0.645	1.000	0.009	
*APEX1*	rs17111750						0.004
	Ca/Co^a^	695/664	354/367	121/110	114/140	75/78	
	OR b	1.00	0.88	2.19	0.78	1.04	
	95%CI	0.85–1.18	0.71–1.10	1.36–3.55	0.52–1.15	0.58–1.86	
	p^c^	0.981	0.271	0.001	0.208	0.897	
	p_ACT_ d	1.000	0.781	0.013	0.573	1.000	
*FEN1*	rs509360	697/665					0.052
	Ca/Co^a^	697/665	353/367	122/111	116/140	74/79	
	OR b	0.93	0.95	1.27	1.09	0.49	
	95%CI	0.78–1.09	0.76–1.19	0.81–2.01	0.71–1.68	0.30–0.79	
	p^c^	0.358	0.662	0.302	0.694	0.003	
	p_ACT_ d	0.888	1.000	0.746	1.000	0.011	
*NTHL1*	rs2516781						0.062
	Ca/Co^a^	688/664	353/363	122/108	115/140	74/77	
	OR b	0.91	1.01	0.83	1.06	0.45	
	95%CI	0.77–1.08	0.80–1.27	0.51–1.34	0.74–1.52	0.25–0.80	
	p^c^	0.271	0.960	0.445	0.747	0.006	
	p_ACT_ d	0.840	1.000	0.960	1.000	0.033	

a Excludes 21 individuals who characterized their race/ethnic group as "other";

b Per-allele ORs and 95%confidence intervals computed using logistic regression assuming a log-additive model and adjusting for age, exam date, sex, clinic, study phase and race among all study participants;

c Crude p-value;

d Crude p-value corrected for multiple comparisons using P_ACT_;

e Race/ethnicity specific per-allele ORs and 95% confidence intervals computed using logistic regression assuming a log-additive model and adjusting for the matching factors age, exam date, sex, clinic, study phase; ^e^ p-value for heterogeneity by race.

When considering stratified analyses by racial/ethnic group, among African-Americans we observed an association with *APEX1* rs17111750 (OR = 2.19; 95%CI = 1.36–3.55; P_ACT_ = 0.013; p_pathway_ = 0.180; p_heterogeneity_ = 0.004)(Table 3). Among Asian-Pacific Islanders we observed an association with two tagSNPs in the *FEN1* gene, rs108499 (OR = 2.12; 95%CI = 1.30–3.45; P_ACT_ = 0.009; p_pathway_ = 0.129; p_heterogeneity_ = 0.024) and rs509360 (OR = 0.40; 95%CI = 0.30–0.79; P_ACT_ = 0.011; p_pathway_ = 0.159; p_heterogeneity_ = 0.052), and one tagSNP in *NTHL1,* rs2516781 (OR = 0.45; 95%CI = 0.25–0.80; P_ACT_ = 0.032; p_pathway_ = 0.461; p_heterogeneity_ = 0.062)(Table 3). All estimates of per-allele associations for adenoma risk among all individuals combined and by race/ethnicity are shown in Table S2.

### BER Genes and Adenoma Risk Taking into Account Adenoma Size and Location

We did not find evidence of any statistically significant per-allele associations within either the small polyp (<1 cm) or large polyp (≥1 cm) group after multiple comparisons adjustment within gene region (P_ACT_). When adenomas location (colon versus rectum), we observed two *NEIL2* tagSNPs were associated with increased risk for rectal adenomas: *NEIL2* rs7015453 (OR = 1.72; 95%CI = 1.24–2.39; P_ACT_ = 0.025; p_heterogeneity_ = 0.003) and rs3757949 (OR = 1.58; 95%CI = 1.18–2.13; P_ACT_ = 0.044; p_heterogeneity_ = 0.004) (Table 4 *).* These two tagSNPs were not found to be in LD among NHW (r^2^ = 0.11), among whom we observed similar findings (data not shown). Complete data for analysis by adenoma location can be found in Table S3.

**Table 4:** Significant BER per-allele associations by polyp sub-site.

		Rectal Polyp						Colon Polyp						Rectal/Colon
Gene	SNP	CA/CO^a^	OR^b^	95%CI	P^c^	P_ACT_ d	P _pathway_ e	CA/CO^a^	OR^b^	95%CI	P^c^	P_ACT_ d	P _pathway_ e	p_het_ f
*NEIL2*	rs3757949	123/669	1.58	1.18–2.13	0.002	0.044	0.610	528/669	1.02	0.85–1.23	0.795	1.000	1.000	0.004
*NEIL2*	rs7015453	123/670	1.72	1.24–2.39	0.001	0.025	0.346	525/670	1.03	0.84–1.27	0.738	1.000	1.000	0.003

a Excludes 21 individuals who characterized their race/ethnic group as "other.";

b Per-allele ORs and 95%confidence intervals computed using multinomial logistic regression assuming a log-additive model and adjusting for age, exam date, sex, clinic, study phase, and race among all study participants;

c Crude p-value;

d Crude p-value corrected for multiple comparisons using P_ACT_;

e Pathway wide (p_pathway_) p-value based on Bonferroni correction of P_ACT_ for the number of BER gene regions investigated (N = 14);

f p-value for heterogeneity by polyp subsite.

### Genetic Variation in BER Genes, Adenoma Risk and Alcohol

Statistically significant interactions were observed for *LIG3* rs1052536 and amount (0 g/day, ≤6 g/day, >6 g/day; interaction p_gene_ = 0.019, interaction p_pathway = _0.241) and frequency of alcohol intake (never, 1 drink/day, >1 drink/day; interaction p_gene_ = 0.029, interaction p_pathway = _0.345) (Table 5). Specifically, among carriers of two major (C) alleles, those who drank more than one drink per day had an 84% increased risk of adenoma compared with non-drinkers (OR = 1.84; 95%CI = 1.09–3.11; p = 0.022; p _for trend_ = 0.024), and those who drank more than 6 grams per day had a 77% increased risk of adenoma compared with non-drinkers (OR = 1.77; 95%CI = 1.17–2.68; p = 0.006; p _for trend_ = 0.003). There was no association between alcohol and adenoma risk for subjects with 1 minor (T) allele (Table 5). When restricting analyses to NHW we observed interactions for *LIG3* rs1052536 that were of similar magnitude although not statistically significant (data not shown).

**Table 5:** Smoking, alcohol, dietary folate and adenoma risk by genotype among all subjects.

Exposure variable	CA/CO	CA/CO	CA/CO	OR a,b,c	95%CI	P	OR a,b,c	95%CI	P	OR a,b,c	95%CI	P
*LIG3* rs105236												
Alcohol Intake	CC	CT	TT	C/C			C/T			T/T		
Never	97/108	99/106	34/30	1^ref^			1^ref^			1^ref^		
≤6 g/day	67/81	73/76	31/38	1.05	0.70–1.58	0.818	0.90	0.67–1.22	0.499	0.77	0.44–1.35	0.369
>6 g/day	89/61	99/99	23/38	1.77	1.17–2.68	0.006	1.01	0.75–1.36	0.962	0.57	0.32–1.01	0.053
p _trend_						0.003			0.719			0.071
				interaction p^d = ^0.005; interaction p_gene_ e = 0.019; interaction p_pathway_ f = 0.241								
**Alcoholic Drinks**	**CC**	**CT**	**TT**	**C/C**			**C/T**			**T/T**		
Never	97/108	99/106	34/30	1^ref^			1^ref^			1^ref^		
≤1 drink/day	112/116	121/117	41/56	1.24	0.86–1.80	0.247	0.96	0.73–1.26	0.752	0.74	0.44–1.23	0.243
>1 drink/day	44/26	51/58	13/20	1.84	1.09–3.11	0.022	0.97	0.67–1.38	0.848	0.51	0.25–1.01	0.052
p _trend_						0.024			0.807			0.048
				interaction p^d = ^0.010; interaction p_gene_ e = 0.029; interaction p_pathway_ f = 0.345								
**Folate mcg/day**	**CC**	**CT**	**TT**	**C/C**			**C/T**			**T/T**		
≤267	106/101	110/92	36/26	1^ref^			1^ref^			1^ref^		
>267–387	65/85	84/89	31/38	0.77	0.52–1.15	0.205	0.74	0.55–0.99	0.040	0.70	0.40–1.23	0.215
≥388	82/64	77/100	21/42	1.29	0.86–1.93	0.212	0.69	0.51–0.93	0.015	0.37	0.20–0.65	0.001
p _trend_						0.175			0.021			<0.001
				interaction p^d = ^0.001; interaction p_gene_ e = 0.006; interaction p_pathway_ f = 0.081								
***LIG3*** ** rs3744358**												
**Folate mcg/day**	**TT**	**TG**	**GG**	**T/T**			**T/G**			**G/G**		
≤267	139/128	91/79	22/12	1^ref^			1^ref^			1^ref^		
>267–387	81/117	74/74	26/19	0.66	0.46–0.94	0.022	0.83	0.59–1.16	0.269	1.05	0.54–2.04	0.890
≥388	103/100	65/80	11/26	1.07	0.75–1.53	0.719	0.63	0.45–0.90	0.010	0.38	0.19–0.75	0.006
p _trend_						0.577			0.010			0.003
				interaction p^d = ^0.008; interaction p_gene_ e = 0.032; interaction p_pathway_ f = 0.451								
***MUTYH*** ** rs10890324**												
**Smoking Pack-yrs**	**AA**	**AG**	**GG**	**A/A**			**A/G**			**G/G**		
Never	116/132	89/108	25/32	1^ref^			1^ref^			1^ref^		
>0–21	75/86	59/74	20/24	0.98	0.67–1.45	0.928	0.99	0.72–1.36	0.950	1.00	0.54–1.86	0.993
>21	95/99	89/64	44/18	1.02	0.70–1.49	0.903	1.76	1.29–2.42	<0.001	3.03	1.67–5.51	<0.001
p _trend_						0.871			<0.001			<0.001
				interaction p^d = ^0.002; interaction p_gene_ e = 0.007; interaction p_pathway_ f = 0.091								
***OGG1*** ** rs159153**												
**Smoking Pack-yrs**	**TT**	**TC**	**CC**	**T/T**			**T/C**			**C/C**		
Never	116/139	96/106	18/27	1^ref^			1^ref^			1^ref^		
>0–21	86/91	52/75	15/16	1.06	0.73–1.55	0.746	0.95	0.67–1.33	0.747	0.84	0.42–1.66	0.615
>21	94/96	109/73	24/12	1.11	0.76–1.62	0.578	1.75	1.26–2.44	0.001	2.76	1.43–5.31	0.002
p _trend_						0.610			<0.001			<0.001
				interaction p^d = ^0.013; interaction p_gene_ e = 0.040; interaction p_pathway_ f = 0.517								
***FEN1*** ** rs108499**												
**Smoking Pack-yrs**	**CC**	**CT**	**TT**	**C/C**			**C/T**			**T/T**		
Never	95/118	87/108	48/44	1^ref^			1^ref^			1^ref^		
>0–21	64/70	56/86	32/27	1.02	0.67–1.54	0.926	0.96	0.71–1.29	0.771	0.90	0.52–1.56	0.7
>21	124/79	82/78	21/22	1.99	1.36–2.91	<0.001	1.25	0.92–1.71	0.156	0.79	0.44–1.41	0.424
p _trend_						<0.001			0.102			0.446
				interaction p^d = ^0.013; interaction p_gene_ e = 0.039; interaction p_pathway_ f = 0.507								

a Odds ratios (ORs) are from models with genotype coded as log-additive (treated as continuous) and adjusted for age, sex, clinic, exam date, study phase, and race among all study participants. Excludes 21 individuals who characterized their race/ethnic group as "other";

b ORs for smoking additionally adjusted for alcohol intake (g/d) and dietary folate intake (mcg/day); ORs for alcohol additionally adjusted for smoking pack-years and dietary folate intake (mcg/d); ORs for dietary folate intake, additionally adjusted for alcohol intake (g/d) and smoking pack-years;

c ORs derived from a common baseline model that included the SNP, smoking, alcohol, dietary folate exposure and interaction terms between genotype and smoking, alcohol, dietary folate exposure levels;

d Crude interaction p-value;

e Within gene region, Bonferroni adjusted interaction p-value, based on the number of SNPs within the respective gene region;

f Pathway wide, Bonferroni adjusted interaction p-value, based on 14 investigated BER gene regions.

### Genetic Variation in BER Genes, Adenoma Risk and Dietary Folate Intake

We observed that the association between folate and adenoma risk was modified by two *LIG3* tagSNPs (rs1052536 interaction p_gene = _0.006, interaction p_pathway = _0.081; rs3744358 interaction p_gene_ = 0.032, interaction p_pathway = _0.451) (Table 5). For each of the two *LIG3* tagSNPs, among individuals with one copy of the minor allele, those with the highest level of dietary folate intake had a 31% and 37% decreased adenoma risk compared to those with the lowest level of dietary folate intake for *LIG3* rs1052536 (OR = 0.61; 95%CI = 0.51–0.93; p = 0.015; p _for trend_ = 0.021) and *LIG3* rs3744358 (OR = 0.63; 95%CI = 0.45–0.90; p = 0.010; p _for trend = _0.003), respectively. Among individuals with two copies of the minor allele, those with the highest level of dietary folate intake had a 72% and 73% decreased adenoma risk compared to those with the lowest level of dietary folate intake for *LIG3* rs1052536 (OR = 0.37; 95%CI = 0.20–0.65; p = 0.0007) and *LIG3* rs3744358 (OR = 0.38; 95%CI = 0.19–0.75; p = 0.006), respectively. Among individuals with 2 copies of the major alleles there was no statistically significant trend across increasing levels of dietary folate intake. When restricting analyses to NHW, similar statistically significant trends were observed for rs1052536 and rs3744358 (data not shown). There is no evidence of LD between *LIG3* tagSNPs rs3744358 and rs*1052536* among NHW.

### Genetic Variation in BER Genes, Adenoma Risk and Smoking

No statistically significant interactions were observed when we considered smoking status (never/quit/current) or smoking duration, after applying within gene Bonferroni corrections. However, we observed that SNPs in three genes modified the association between smoking pack-years and adenoma risk, with statistically significant interaction tests that survived within gene Bonferroni correction. First, we observed smoking pack-years was modified by *MUTYH* rs10890324 (interaction p_gene = _0.007, interaction p_pathway = _0.091) (Table 5). Whereas among individuals with two copies of the major (A) allele there was no association between increasing pack-years and adenoma risk (p _for trend_ = 0.871), among carriers of one copy of the minor (G) allele, having smoked over 21 pack-years was associated with a 76% increased adenoma risk when compared to never smokers (OR = 1.76; 95%CI = 1.29–2.42; p<0.001; p _for trend_ <0.001). Among carriers of two minor (G) alleles, smoking more than 21 pack-years was associated with a 3-fold increased risk of adenomas (p _for trend_ <0.001). Analysis among non-Hispanic Whites was not performed due to sparse data.

Second, the *OGG1* rs159153 SNP modified the association between smoking pack-years and adenoma risk (interaction p_gene = _0.040, interaction p_pathway = _0.517). While among individuals with two copies of the major (A) allele there was no association between increasing pack-years and adenoma risk (p _for trend_ = 0.610), among carriers of one copy of the minor (C) allele, having smoked over 21 pack-years was associated with a 75% increased adenoma risk when compared to never smokers (OR = 1.75; 95%CI = 1.26–2.44; p = 0.001; p _for trend_ <0.001). Among individuals carrying a second minor (C) allele, smoking over 21 pack-years was significantly associated with an almost 3-fold increased risk of adenoma (p _for trend_ <0.001) when compared to never smokers. Significant trends of increasing adenoma risk among carriers of 1 and 2 minor alleles were observed with increasing smoking years, but there was no statistically significant evidence of interaction between *OGG1* rs159153 and smoking years and adenoma risk.

Finally, the *FEN1* rs108499 SNP also modified the association between smoking pack-years and adenoma risk (interaction p_gene = _0.039, interaction p_pathway = _0.507). Among individuals with two copies of the major (C) allele smoking more than 21 pack-years was associated with a 2-fold increased risk of adenomas compared to never smokers (p _for trend_ <0.001) but there was no association among individuals with either one copy of the minor (T) allele or two copies of the minor (T) allele (Table 5). While a similar significant trend of increasing adenoma risk was observed with increasing smoking years, there was no statistically significant evidence of interaction. Analyses among NHW only were not performed due to sparse data.

## Discussion

We investigated potential associations between 182 tagSNPs from 14 BER gene regions and their role in modifying the effects of smoking, dietary folate intake and alcohol consumption on colorectal adenoma risk. We observed statistically significant associations between colorectal adenoma risk and polymorphisms in the *FEN1* gene (two tagSNPs) and *NTHL1* gene (one tagSNP) among Asian-Pacific Islanders, and the *APEX1* gene among African-Americans (one tagSNP). Significant associations were also observed for two unlinked tagSNPs in the *NEIL2* gene and rectal adenoma risk. None of the six tagSNPs were found to modify the effects of dietary folate, or alcohol on adenoma risk. However, one of these six tagSNPs, *FEN1* rs108499, was found to modify the effects of smoking on adenoma risk. Moreover, we observed evidence that SNPs in other BER genes modified the effect of smoking *(MUTYH, OGG1)*, alcohol *(LIG3)*, and dietary folate *(LIG3)* on colorectal adenoma risk. Overall, our findings support the hypothesis that oxidative damage induced by these exposures may play an important role in colorectal adenoma development.

Previous studies have investigated BER gene polymorphisms and adenoma risk [25], [26], [34], [35], [36], [37], [38]. With one exception [38], they have been limited to a handful of candidate SNPs within *XRCC1, PARP1, OGG1,* and *APEX* genes. Even fewer considered the modifier role of alcohol, smoking or dietary folate intake [26], [37], [38]. Therefore, to our knowledge, this is the first comprehensive examination of the BER pathway and colorectal adenoma risk taking into account relevant environmental risk factors.

We report a two-fold increased adenoma risk associated with SNP rs17111750 located 5′-upstream of APEX1, only among African-Americans. The human *APEX1* protein is an apurinic/apyrimidinic endonuclease that repairs DNA damage caused by oxidative and alkylating agents [39]. A previous study has reported a positive association between this SNP and colorectal adenoma, and another one reported lack of association with prostate cancer risk [38], [40]. Among African-Americans *APEX1* rs17111750 is not in LD (r^2^<0.05) with two other *APEX1* SNPs, rs1048945 and rs1130409, previously reported by us and others to be associated with CRC and adenoma risk [37], [41], [42], [43].

We observed that the minor alleles of the *FEN1* SNPs rs509360 and rs108499 were associated with a two-fold increased and decreased risk of adenoma, respectively, among Asian-Pacific Islanders. Moreover, among all subjects combined, rs108499 was found to modify the effect of smoking on adenoma risk. Both rs509360 and rs108499 are located 5′-upstream of *FEN1*, occurring within intronic regions of *C11orf9*. A previous study among predominantly NHW found no associations between these *FEN1* SNPs and colorectal adenoma [38]. The *FEN1* endonuclease is involved in BER and DNA replication and has been reported to play important roles in genomic stability [44], chronic inflammation, autoimmunity and cancers [45], [46]. In two studies conducted among Chinese populations, polymorphisms in the *FEN1* promoter and 3′-UTR were associated with reduced *FEN1* expression, increased DNA damage and increased risks for lung and CRC [47], [48].

The *NTHL1* rs2516781 SNP was associated with a two-fold decreased risk of adenoma only among Asian-Pacific Islanders. The NTHL1 protein has DNA N-glycosylase activity as well as apurinic and/or apyrimidinic endonuclease activity [49], [50], [51]. The rs2516781 SNP is 3′-downstream of the *NTHL1* gene, an intronic SNP within the solute carrier family 9 (sodium/hydrogen exchanger), member 3 regulator 2 (*SLC9A3R2*) gene. *SLC9A3R2* is involved in regulation of SLC9A3, the sodium/hydrogen exchanger involved in intestinal sodium absorption [52]. This SNP has not been previously assessed in studies of colorectal adenoma or CRC.

Two *NEIL2* SNPs, rs11785481 and rs3757949, were associated with risk for rectal adenomas. Among NHW these two SNPs are not in LD (r^2^<0.1). The NEIL2 protein has DNA glycosylase activity and apurinic/apyrimidinic endonuclease activity [39]. The rs3757949 SNP, is an intronic SNP located within *GATA4,* a gene that codes for a transcription factor relevant for gene expression in gastrointestinal epithelium [53], [54]. Promoter hyper-methylation and the subsequent transcriptional silencing of *GATA4* are commonly seen in CRC cell lines and primary CRCs [55].

We found evidence that the *MUTYH* rs10890324 SNP, which flanks the 3′-end of *MUTYH*, modified the association between smoking and adenoma risk. The *MUTYH* gene encodes a DNA glycosylase, and germline mutations in highly conserved residues of the *MUTYH* gene predispose individuals to *MUTYH*-associated polyposis coli [56] as well as sporadic CRC [57], [58]. We also found that the *OGG1* rs159153 SNP, located 5′-upstream of *OGG1*, modified the association between smoking and adenoma risk. The 8-oxoguanine glycosylase 1 (*OGG1)* enzyme can excise the highly mutagenic 8-oxoguanine lesions induced by ROS and normal cellular metabolism [59]. The more commonly investigated *OGG1* Ser326Cys SNP (rs1052133) is not in LD with rs159153 among NHW (r^2^ = 0.08).

Finally, we found evidence that the *LIG3* rs1052536 SNP modified the association between alcohol intake and adenoma risk. In addition, *LIG3* SNPs rs1052536 and rs3744358 modified the association between dietary folate intake and adenoma risk. *LIG3*, a ligase involved in maintaining genomic integrity, encodes proteins present in the mitochondria and nucleus.

Our study had several strengths, including the relatively large sample size and the use of a comprehensive tagSNP approach to thoroughly investigate genetic variation in 14 BER genes. Among the weaknesses of our study is the fact that results are only applicable to the sigmoid colon and rectum, and the fact that cases had colonoscopy performed but not controls; therefore, some controls might have had more proximal polyps out of reach of sigmoidscope [60]. Even though we conducted a large number of tests as part of our analyses, we believe our approach for multiple comparisons adjustment in the SNP main effect analyses and the gene environment analyses is a conservative method of reducing the number of false positive results.

In conclusion, our findings suggest that genetic variation in non-coding BER gene regions can modify the risk of adenoma, particularly in combination with key environmental exposures. These findings highlight a relevant role for oxidative damage induced by these environmental exposures in colorectal adenoma development.

## Supporting Information

Table S1 **Genes/SNPs included in study and minor allele frequencies.**
(XLSX)

Table S2 **Single SNP analysis for entire study population and by race/ethnic group.**
(XLSX)

Table S3 **Per allele associations by polyp subsite.**
(XLSX)

## References

[1] ACS (2011) Colorectal Cancer Facts & Figures 2011–2013. Atlanta: American Cancer Society.

[2] Stewart SL, Wike JM, Kato I, Lewis DR, Michaud F (2006) A population-based study of colorectal cancer histology in the United States, 1998–2001. Cancer 107: 1128–1141. 16802325 10.1002/cncr.22010

[3] Goroll AH, Mulley AG (2009) Primary care medicine : office evaluation and management of the adult patient. Philadelphia: Wolters Kluwer/Lippincott Williams & Wilkins. xxix, 1613 p., 1616 p. of plates p.

[4] Atkin WS, Saunders BP (2002) Surveillance guidelines after removal of colorectal adenomatous polyps. Gut 51 Suppl 5V6–9. 12221031 10.1136/gut.51.suppl_5.v6 PMC1867736

[5] Winawer SJ, Zauber AG, Ho MN, O'Brien MJ, Gottlieb LS, et al (1993) Prevention of colorectal cancer by colonoscopic polypectomy. The National Polyp Study Workgroup. The New England journal of medicine 329: 1977–1981. 8247072 10.1056/NEJM199312303292701

[6] Winawer SJ (2001) A quarter century of colorectal cancer screening: progress and prospects. Journal of clinical oncology : official journal of the American Society of Clinical Oncology 19: 6S–12S. 11560964

[7] Terry MB, Neugut AI, Bostick RM, Sandler RS, Haile RW, et al (2002) Risk factors for advanced colorectal adenomas: a pooled analysis. Cancer epidemiology, biomarkers & prevention : a publication of the American Association for Cancer Research, cosponsored by the American Society of Preventive Oncology 11: 622–629. 12101109

[8] Botteri E, Iodice S, Raimondi S, Maisonneuve P, Lowenfels AB (2008) Cigarette smoking and adenomatous polyps: a meta-analysis. Gastroenterology 134: 388–395. 18242207 10.1053/j.gastro.2007.11.007

[9] Duthie SJ, Narayanan S, Sharp L, Little J, Basten G, et al (2004) Folate, DNA stability and colo-rectal neoplasia. The Proceedings of the Nutrition Society 63: 571–578. 15831129 10.1079/pns2004

[10] Mason JB (2009) Folate, cancer risk, and the Greek god, Proteus: a tale of two chameleons. Nutrition reviews 67: 206–212. 19335714 10.1111/j.1753-4887.2009.00190.x PMC2763118

[11] Pryor WA (1997) Cigarette smoke radicals and the role of free radicals in chemical carcinogenicity. Environmental health perspectives 105 Suppl 4875–882. 9255574 10.1289/ehp.97105s4875 PMC1470037

[12] Friedberg EC, ebrary Inc. (2006) DNA repair and mutagenesis. 2nd ed. Washington, D.C.: ASM Press,. pp. xxix, 1118 p. ill. (some col.) 1129 cm.

[13] Mannino DM, Mulinare J, Ford ES, Schwartz J (2003) Tobacco smoke exposure and decreased serum and red blood cell folate levels: data from the Third National Health and Nutrition Examination Survey. Nicotine & tobacco research : official journal of the Society for Research on Nicotine and Tobacco 5: 357–362. 12791531 10.1080/1462220031000094330

[14] Blount BC, Mack MM, Wehr CM, MacGregor JT, Hiatt RA, et al (1997) Folate deficiency causes uracil misincorporation into human DNA and chromosome breakage: implications for cancer and neuronal damage. Proceedings of the National Academy of Sciences of the United States of America 94: 3290–3295. 9096386 10.1073/pnas.94.7.3290 PMC20362

[15] Secretan B, Straif K, Baan R, Grosse Y, El Ghissassi F, et al (2009) A review of human carcinogens–Part E: tobacco, areca nut, alcohol, coal smoke, and salted fish. The lancet oncology 10: 1033–1034. 19891056 10.1016/s1470-2045(09)70326-2

[16] American Institute for Cancer Research, World Cancer Research Fund (2007) Food, nutrition, physical activity and the prevention of cancer : a global perspective : a project of World Cancer Research Fund International. Washington, D.C.: American Institute for Cancer Research. xxv, 517 p. p.

[17] Baron JA, Sandler RS, Haile RW, Mandel JS, Mott LA, et al (1998) Folate intake, alcohol consumption, cigarette smoking, and risk of colorectal adenomas. Journal of the National Cancer Institute 90: 57–62. 9428784 10.1093/jnci/90.1.57

[18] Seitz HK, Simanowski UA, Garzon FT, Rideout JM, Peters TJ, et al (1990) Possible role of acetaldehyde in ethanol-related rectal cocarcinogenesis in the rat. Gastroenterology 98: 406–413. 2295396 10.1016/0016-5085(90)90832-l

[19] Poschl G, Seitz HK (2004) Alcohol and cancer. Alcohol and alcoholism 39: 155–165. 15082451 10.1093/alcalc/agh057

[20] Kune GA, Vitetta L (1992) Alcohol consumption and the etiology of colorectal cancer: a review of the scientific evidence from 1957 to 1991. Nutrition and cancer 18: 97–111. 1437657 10.1080/01635589209514210

[21] Brooks PJ, Theruvathu JA (2005) DNA adducts from acetaldehyde: implications for alcohol-related carcinogenesis. Alcohol 35: 187–193. 16054980 10.1016/j.alcohol.2005.03.009

[22] Seitz HK, Maurer B, Stickel F (2005) Alcohol consumption and cancer of the gastrointestinal tract. Digestive diseases 23: 297–303. 16508294 10.1159/000090177

[23] Das SK, Vasudevan DM (2007) Alcohol-induced oxidative stress. Life sciences 81: 177–187. 17570440 10.1016/j.lfs.2007.05.005

[24] Robertson AB, Klungland A, Rognes T, Leiros I (2009) DNA repair in mammalian cells: Base excision repair: the long and short of it. Cellular and molecular life sciences : CMLS 66: 981–993. 19153658 10.1007/s00018-009-8736-z PMC11131461

[25] Stern MC, Siegmund KD, Corral R, Haile RW (2005) XRCC1 and XRCC3 polymorphisms and their role as effect modifiers of unsaturated fatty acids and antioxidant intake on colorectal adenomas risk. Cancer Epidemiol Biomarkers Prev 14: 609–615. 15767338 10.1158/1055-9965.EPI-04-0189

[26] Stern MC, Siegmund KD, Conti DV, Corral R, Haile RW (2006) XRCC1, XRCC3, and XPD polymorphisms as modifiers of the effect of smoking and alcohol on colorectal adenoma risk. Cancer Epidemiol Biomarkers Prev 15: 2384–2390. 17164360 10.1158/1055-9965.EPI-06-0381

[27] Lin HJ, Probst-Hensch NM, Ingles SA, Han CY, Lin BK, et al (1995) Glutathione transferase (GSTM1) null genotype, smoking, and prevalence of colorectal adenomas. Cancer Res 55: 1224–1226. 7882312

[28] Longnecker MP, Chen MJ, Probst-Hensch NM, Harper JM, Lee ER, et al (1996) Alcohol and smoking in relation to the prevalence of adenomatous colorectal polyps detected at sigmoidoscopy. Epidemiology 7: 275–280. 8728441 10.1097/00001648-199605000-00010

[29] Haile RW, Witte JS, Longnecker MP, Probst-Hensch N, Chen MJ, et al (1997) A sigmoidoscopy-based case-control study of polyps: macronutrients, fiber and meat consumption. Int J Cancer 73: 497–502. 9389562 10.1002/(sici)1097-0215(19971114)73:4<497::aid-ijc7>3.0.co;2-v

[30] Barrett JC, Fry B, Maller J, Daly MJ (2005) Haploview: analysis and visualization of LD and haplotype maps. Bioinformatics 21: 263–265. 15297300 10.1093/bioinformatics/bth457

[31] Levine AJ, Lee W, Figueiredo JC, Conti DV, Vandenberg DJ, et al (2011) Variation in folate pathway genes and distal colorectal adenoma risk: a sigmoidoscopy-based case-control study. Cancer Causes Control 22: 541–552. 21274745 10.1007/s10552-011-9726-7 PMC3059778

[32] Conneely KN, Boehnke M (2007) So many correlated tests, so little time! Rapid adjustment of P values for multiple correlated tests. Am J Hum Genet 81: 1158–1168. 17966093 10.1086/522036 PMC2276357

[33] Storey JD, Tibshirani R (2003) Statistical significance for genomewide studies. Proceedings of the National Academy of Sciences of the United States of America 100: 9440–9445. 12883005 10.1073/pnas.1530509100 PMC170937

[34] Hansen R, Saebo M, Skjelbred CF, Nexo BA, Hagen PC, et al (2005) GPX Pro198Leu and OGG1 Ser326Cys polymorphisms and risk of development of colorectal adenomas and colorectal cancer. Cancer letters 229: 85–91. 15946795 10.1016/j.canlet.2005.04.019

[35] Skjelbred CF, Saebo M, Wallin H, Nexo BA, Hagen PC, et al (2006) Polymorphisms of the XRCC1, XRCC3 and XPD genes and risk of colorectal adenoma and carcinoma, in a Norwegian cohort: a case control study. BMC cancer 6: 67. 16542436 10.1186/1471-2407-6-67 PMC1458350

[36] Gsur A, Bernhart K, Baierl A, Feik E, Fuhrlinger G, et al (2011) No association of XRCC1 polymorphisms Arg194Trp and Arg399Gln with colorectal cancer risk. Cancer epidemiology 35: e38–41. 21612998 10.1016/j.canep.2011.03.005

[37] Berndt SI, Huang WY, Fallin MD, Helzlsouer KJ, Platz EA, et al (2007) Genetic variation in base excision repair genes and the prevalence of advanced colorectal adenoma. Cancer research 67: 1395–1404. 17283177 10.1158/0008-5472.CAN-06-1390

[38] Gao Y, Hayes RB, Huang WY, Caporaso NE, Burdette L, et al (2011) DNA repair gene polymorphisms and tobacco smoking in the risk for colorectal adenomas. Carcinogenesis 32: 882–887. 21504893 10.1093/carcin/bgr071 PMC3106441

[39] Simonelli V, Mazzei F, D'Errico M, Dogliotti E (2012) Gene susceptibility to oxidative damage: From single nucleotide polymorphisms to function. Mutation research 731: 1–13. 22155132 10.1016/j.mrfmmm.2011.10.012

[40] Barry KH, Koutros S, Berndt SI, Andreotti G, Hoppin JA, et al (2011) Genetic variation in base excision repair pathway genes, pesticide exposure, and prostate cancer risk. Environmental health perspectives 119: 1726–1732. 21810555 10.1289/ehp.1103454 PMC3261977

[41] Brevik A, Joshi AD, Corral R, Onland-Moret NC, Siegmund KD, et al (2010) Polymorphisms in base excision repair genes as colorectal cancer risk factors and modifiers of the effect of diets high in red meat. Cancer Epidemiol Biomarkers Prev 19: 3167–3173. 21037106 10.1158/1055-9965.EPI-10-0606 PMC3058341

[42] Kasahara M, Osawa K, Yoshida K, Miyaishi A, Osawa Y, et al (2008) Association of MUTYH Gln324His and APEX1 Asp148Glu with colorectal cancer and smoking in a Japanese population. Journal of experimental & clinical cancer research : CR 27: 49. 18823566 10.1186/1756-9966-27-49 PMC2575194

[43] Pardini B, Naccarati A, Novotny J, Smerhovsky Z, Vodickova L, et al (2008) DNA repair genetic polymorphisms and risk of colorectal cancer in the Czech Republic. Mutation research 638: 146–153. 17991492 10.1016/j.mrfmmm.2007.09.008

[44] Chapados BR, Hosfield DJ, Han S, Qiu J, Yelent B, et al (2004) Structural basis for FEN-1 substrate specificity and PCNA-mediated activation in DNA replication and repair. Cell 116: 39–50. 14718165 10.1016/s0092-8674(03)01036-5

[45] Zheng L, Dai H, Zhou M, Li M, Singh P, et al (2007) Fen1 mutations result in autoimmunity, chronic inflammation and cancers. Nature medicine 13: 812–819. 10.1038/nm1599 17589521

[46] Kucherlapati M, Yang K, Kuraguchi M, Zhao J, Lia M, et al (2002) Haploinsufficiency of Flap endonuclease (Fen1) leads to rapid tumor progression. Proceedings of the National Academy of Sciences of the United States of America 99: 9924–9929. 12119409 10.1073/pnas.152321699 PMC126601

[47] Yang M, Guo H, Wu C, He Y, Yu D, et al (2009) Functional FEN1 polymorphisms are associated with DNA damage levels and lung cancer risk. Human mutation 30: 1320–1328. 19618370 10.1002/humu.21060

[48] Liu L, Zhou C, Zhou L, Peng L, Li D, et al (2012) Functional FEN1 genetic variants contribute to risk of hepatocellular carcinoma, esophageal cancer, gastric cancer and colorectal cancer. Carcinogenesis 33: 119–123. 22072618 10.1093/carcin/bgr250

[49] Luna L, Bjoras M, Hoff E, Rognes T, Seeberg E (2000) Cell-cycle regulation, intracellular sorting and induced overexpression of the human NTH1 DNA glycosylase involved in removal of formamidopyrimidine residues from DNA. Mutation research 460: 95–104. 10882850 10.1016/s0921-8777(00)00015-x

[50] Aspinwall R, Rothwell DG, Roldan-Arjona T, Anselmino C, Ward CJ, et al (1997) Cloning and characterization of a functional human homolog of Escherichia coli endonuclease III. Proceedings of the National Academy of Sciences of the United States of America 94: 109–114. 8990169 10.1073/pnas.94.1.109 PMC19249

[51] Ikeda S, Biswas T, Roy R, Izumi T, Boldogh I, et al (1998) Purification and characterization of human NTH1, a homolog of Escherichia coli endonuclease III. Direct identification of Lys-212 as the active nucleophilic residue. The Journal of biological chemistry 273: 21585–21593. 9705289 10.1074/jbc.273.34.21585

[52] Yun CH, Oh S, Zizak M, Steplock D, Tsao S, et al (1997) cAMP-mediated inhibition of the epithelial brush border Na+/H+ exchanger, NHE3, requires an associated regulatory protein. Proceedings of the National Academy of Sciences of the United States of America 94: 3010–3015. 9096337 10.1073/pnas.94.7.3010 PMC20313

[53] Temsah R, Nemer M (2005) GATA factors and transcriptional regulation of cardiac natriuretic peptide genes. Regulatory peptides 128: 177–185. 15837526 10.1016/j.regpep.2004.12.026

[54] Molkentin JD (2000) The zinc finger-containing transcription factors GATA-4, -5, and -6. Ubiquitously expressed regulators of tissue-specific gene expression. The Journal of biological chemistry 275: 38949–38952. 11042222 10.1074/jbc.R000029200

[55] Zheng R, Blobel GA (2010) GATA Transcription Factors and Cancer. Genes & cancer 1: 1178–1188. 21779441 10.1177/1947601911404223 PMC3092280

[56] Cheadle JP, Sampson JR (2007) MUTYH-associated polyposis–from defect in base excision repair to clinical genetic testing. DNA repair 6: 274–279. 17161978 10.1016/j.dnarep.2006.11.001

[57] Farrington SM, Tenesa A, Barnetson R, Wiltshire A, Prendergast J, et al (2005) Germline susceptibility to colorectal cancer due to base-excision repair gene defects. American journal of human genetics 77: 112–119. 15931596 10.1086/431213 PMC1226182

[58] Cleary SP, Cotterchio M, Jenkins MA, Kim H, Bristow R, et al (2009) Germline MutY human homologue mutations and colorectal cancer: a multisite case-control study. Gastroenterology 136: 1251–1260. 19245865 10.1053/j.gastro.2008.12.050 PMC2739726

[59] Parsons JL, Zharkov DO, Dianov GL (2005) NEIL1 excises 3′ end proximal oxidative DNA lesions resistant to cleavage by NTH1 and OGG1. Nucleic acids research 33: 4849–4856. 16129732 10.1093/nar/gki816 PMC1196207

[60] Foutch PG, Mai H, Pardy K, DiSario JA, Manne RK, et al (1991) Flexible sigmoidoscopy may be ineffective for secondary prevention of colorectal cancer in asymptomatic, average-risk men. Digestive diseases and sciences 36: 924–928. 2070706 10.1007/BF01297142

